# Recruiting Young Gay and Bisexual Men for a Human Papillomavirus Vaccination Intervention Through Social Media: The Effects of Advertisement Content

**DOI:** 10.2196/publichealth.7545

**Published:** 2017-06-02

**Authors:** Paul L Reiter, Mira L Katz, Jose A Bauermeister, Abigail B Shoben, Electra D Paskett, Annie-Laurie McRee

**Affiliations:** ^1^ College of Medicine Ohio State University Columbus, OH United States; ^2^ College of Public Health Ohio State University Columbus, OH United States; ^3^ Department of Family and Community Health University of Pennsylvania School of Nursing Philadelphia, PA United States; ^4^ Division of General Pediatrics and Adolescent Health University of Minnesota Medical School Minneapolis, MN United States

**Keywords:** HPV, HPV vaccine, gay and bisexual men, social media

## Abstract

**Background:**

Web-based approaches, specifically social media sites, represent a promising approach for recruiting young gay and bisexual men for research studies. Little is known, however, about how the performance of social media advertisements (ads) used to recruit this population is affected by ad content (ie, image and text).

**Objective:**

The aim of this study was to evaluate the effects of different images and text included in social media ads used to recruit young gay and bisexual men for the pilot test of a Web-based human papillomavirus (HPV) vaccination intervention.

**Methods:**

In July and September 2016, we used paid Facebook advertisements to recruit men who were aged 18-25 years, self-identified as gay or bisexual, US resident, and had not received HPV vaccine. A 4x2x2 factorial experiment varied ad image (a single young adult male, a young adult male couple, a group of young adult men, or a young adult male talking to a doctor), content focus (text mentioning HPV or HPV vaccine), and disease framing (text mentioning cancer or a sexually transmitted disease [STD]). Poisson regression determined whether these experimental factors affected ad performance.

**Results:**

The recruitment campaign reached a total of 35,646 users who viewed ads for 36,395 times. This resulted in an overall unique click-through rate of 2.01% (717/35,646) and an overall conversion rate of 0.66% (241/36,395). Reach was higher for ads that included an image of a couple (incidence rate ratio, IRR=4.91, 95% CI 2.68-8.97, *P*<.001) or a group (IRR=2.65, 95% CI 1.08-6.50, *P*=.03) compared with those that included an image of a single person. Ads that included an image of a couple also had a higher conversion rate (IRR=2.56, 95% CI 1.13-5.77, *P*=.02) than ads that included an image of a single person. Ads with text mentioning an STD had a higher unique click-through rate compared with ads with text mentioning cancer (IRR=1.34, 95% CI 1.06-1.69, *P*=.01). The campaign cost a total of US $413.72 and resulted in 150 eligible and enrolled individuals (US $2.76 per enrolled participant).

**Conclusions:**

Facebook ads are a convenient and cost-efficient strategy for reaching and recruiting young gay and bisexual men for a Web-based HPV vaccination intervention. To help optimize ad performance among this population, researchers should consider the importance of the text and image included in the social media recruitment ads.

## Introduction

Human papillomavirus (HPV) infection is the most common sexually transmitted infection in the United States [1]. Gay and bisexual men have high rates of HPV infection and several HPV-related diseases including anal cancer and genital warts [2-4]. The Advisory Committee on Immunization Practices (ACIP) currently recommends routine HPV vaccination for males aged 11-12 years in the United States, with catch-up vaccination for ages 13-21 years [5]. Importantly, the ACIP also recommends routine HPV vaccination for men who have sex with men, including those who identify as gay or bisexual or who intend to have sex with men, through the age of 26 years [5]. Two doses of HPV vaccine are now recommended if the vaccine series is initiated before turning 15 years old, whereas 3 doses are recommended if the vaccine series is initiated after turning 15 years old [5]. Despite recommendations, current HPV vaccine coverage remains modest among males in the United States, including among young gay and bisexual men [6-9]. Recent studies suggest that fewer than 15% of young gay and bisexual men have received any doses of the HPV vaccine [7-9].

Efforts are therefore needed to increase HPV vaccination among young gay and bisexual men. However, reaching and recruiting this population may pose a challenge to HPV vaccination interventions. Recruitment via community settings (eg, community events and organizations) has been a frequently used approach for recruiting gay and bisexual men into research studies, but it is an approach that may oversample individuals who are more involved with the lesbian, gay, bisexual, and transgender (LGBT) community [10]. Some individuals may also be less willing to self-identify as gay or bisexual (or disclose same-sex behavior or attraction) when recruited in-person compared with other approaches [11].

Web-based approaches, specifically social media sites, represent a newer and promising recruitment strategy [12,13]. Facebook is the most popular social media site among US adults, with an estimated 72% of adult Internet users reporting use of Facebook [14]. With more than 6 million sexual minority users in the United States [15], Facebook is a promising platform for recruiting young gay and bisexual men. Several recent efforts have successfully used Facebook to recruit young adults including sexual minority young adults, into research studies for a range of health-related topics [16-25]. Several of these studies reported that Facebook was a convenient and cost-efficient strategy for recruiting young adults [17,18,20,22].

The content of the recruitment ads likely plays a role in the success of a Facebook recruitment campaign. Indeed, past studies involving smoking cessation and mental health have shown that both the images and text included in Facebook ads affect ad performance and recruitment metrics (eg, number of Facebook users who click on an ad) [26,27]. Little is known, however, about how Facebook ad content (ie, image and text) affects the success of recruitment campaigns for sexual minority populations. We report the results of an experiment to determine how Facebook ad content affected the recruitment of young gay and bisexual men for the pilot test of an HPV vaccination intervention. Results will be highly useful for planning and implementing future interventions among this population.

## Methods

### Study Overview

“Outsmart HPV” is a mobile-friendly Web-based HPV vaccination intervention developed for young gay and bisexual men. Men were eligible for this project if they (1) were in the age range of 18-25 years, (2) self-identified as gay or bisexual, (3) were a US resident, and (4) had not received any doses of HPV vaccine. Following intervention development, we conducted a pilot test of this project. Recruitment for the pilot test occurred in two waves, with the first wave occurring in July 2016 and the second in September 2016. All recruitment occurred via paid Facebook ads. This report includes recruitment results from the pilot test. The Institutional Review Board at The Ohio State University approved this study. The parent randomized trial is registered at ClinicalTrials.gov (identifier NCT02835755).

### Facebook Advertisements

We created recruitment ads using Facebook’s Ads Manager program. All ads included a headline, main text, image, Outsmart HPV project logo, and weblink to the project website. Ads adhered to Facebook’s requirements at the time of ad development, including character limits (25 characters for the headline and 90 characters for the main text) and image restrictions (any image used could not include more than 20% text) [28]. We designed all ads to appear in the “News Feed” on Facebook, which is a streaming list of updates from the user’s connections (eg, friends) and advertisers. We focused solely on News Feed ads, as opposed to other Facebook ad locations (eg, right column), since News Feed ads are more effective in terms of recruitment metrics for research studies [26].

**Figure 1:**
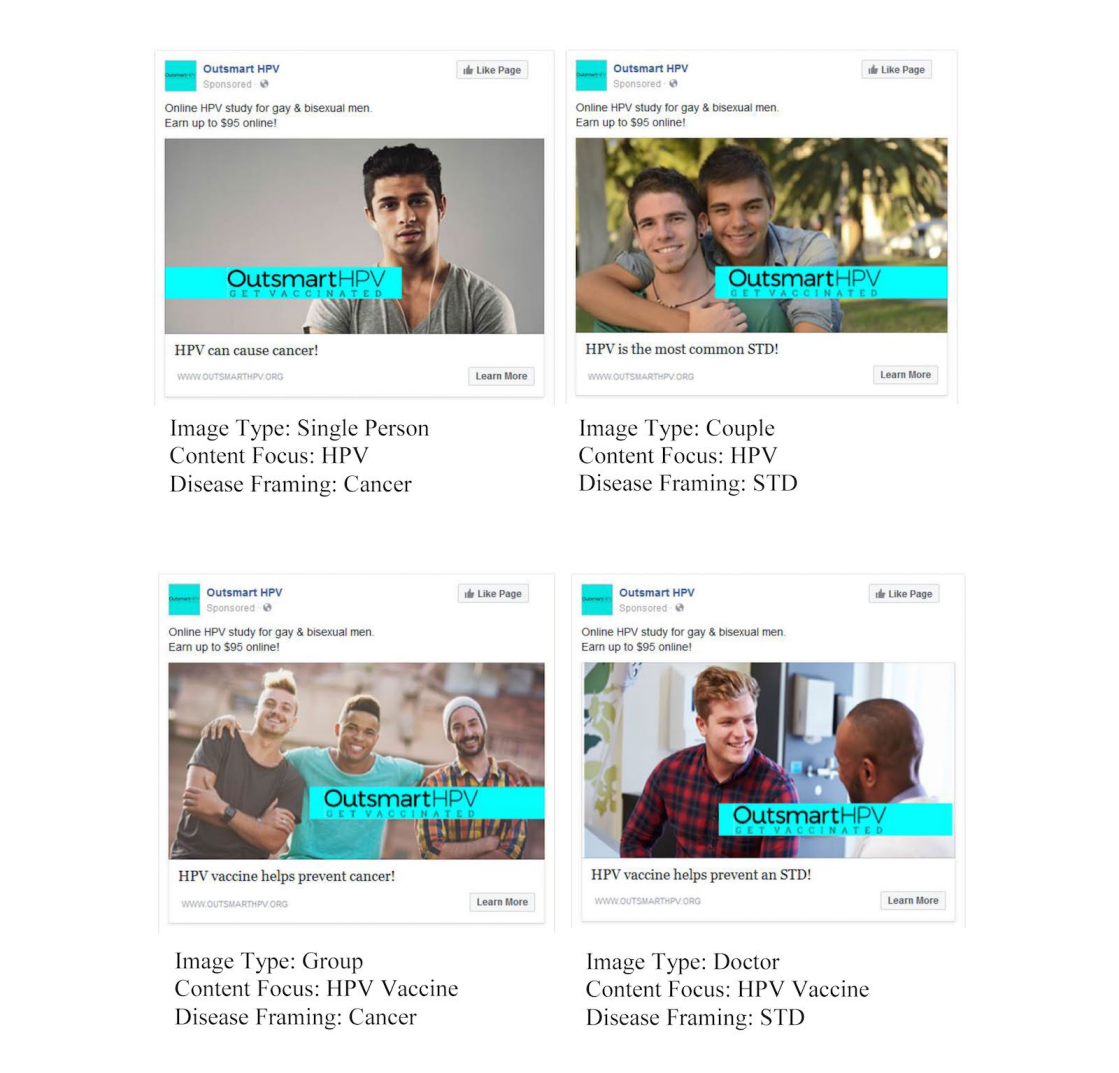
Example Facebook recruitment ads for Outsmart HPV (human papillomavirus). All ads were News Feed ads, and experimental factors included: image type (single person, couple, group, or doctor), content focus (HPV or HPV vaccine), and disease framing (cancer or sexually transmitted disease [STD]).

The headline (“Earn Up To $95 Online!”), Outsmart HPV logo, and weblink to the project website were identical for all ads. The main text for all ads started with “Online HPV study for gay & bisexual men,” but the remaining main text and image varied across the ads using a 4x2x2 factorial experiment design. Experimental factors included image type, content focus, and disease framing. Image type (4 conditions) refers to the primary image featured in the ad. All ad images featured at least one male who appeared to be a young adult in the target age group, but ads varied on the number and type of people in the image: (1) a single man (“single person”), (2) a male couple (“couple”), (3) a group of more than 2 men (“group”), or (4) a man interacting with a health care provider (“doctor”). The people included in the images were from a range of racial or ethnic groups. Content focus (2 conditions) involved whether the ad’s remaining main text mentioned (1) HPV, or (2) HPV vaccine. Disease framing (2 conditions) involved whether the ad’s remaining main text mentioned (1) cancer, or (2) a sexually transmitted disease (STD). We created 2 ads for each of the 16 experimental conditions (ie, 2 variations of each image type), resulting in a total of 32 ads. Figure 1 shows example ads across various experimental conditions. Facebook approved all ads before their use.

### Recruitment Campaign

We targeted all ads to Facebook users by sex (male), age (18-25 years), location (United States), and language (English). We further targeted the ads on keywords generated from information that Facebook users add to their Timeline, pages they “like,” or ads they have previously clicked on. The keywords used in our campaign included “bisexuality,” “homosexuality,” “same-sex relationship,” “genderqueer,” “gay pride,” “LGBT community,” “LGBT culture,” or “rainbow flag (LGBT movement).” The chance that a given ad appeared on a user’s page was determined by a Facebook algorithm that considers several factors (eg, the spending limit specified for the ad, competition from other ads).

### Project Enrollment

Ads linked potential participants to the project website or the project’s Facebook page (which in turn directed potential participants to the project website). We collaborated with the Center for Health Communications Research (CHCR) at the University of Michigan to develop and maintain the project website, which was a mobile-friendly website accessible by desktop or laptop, tablet computer, or smartphone (iOS and Android). Once on the project website, potential participants first completed a project eligibility screener. Those determined to be eligible were then asked to provide informed consent and create a project account. Following account creation, participants completed a preintervention Web-based survey, were randomly assigned to either the intervention or control arm, and then completed remaining study activities (eg, viewing intervention or control materials on the Web about HPV vaccine and completing 3 additional Web-based surveys over the course of 7 months). Participants could earn up to US $95 in gift cards during the course of the project.

**Figure 2:**
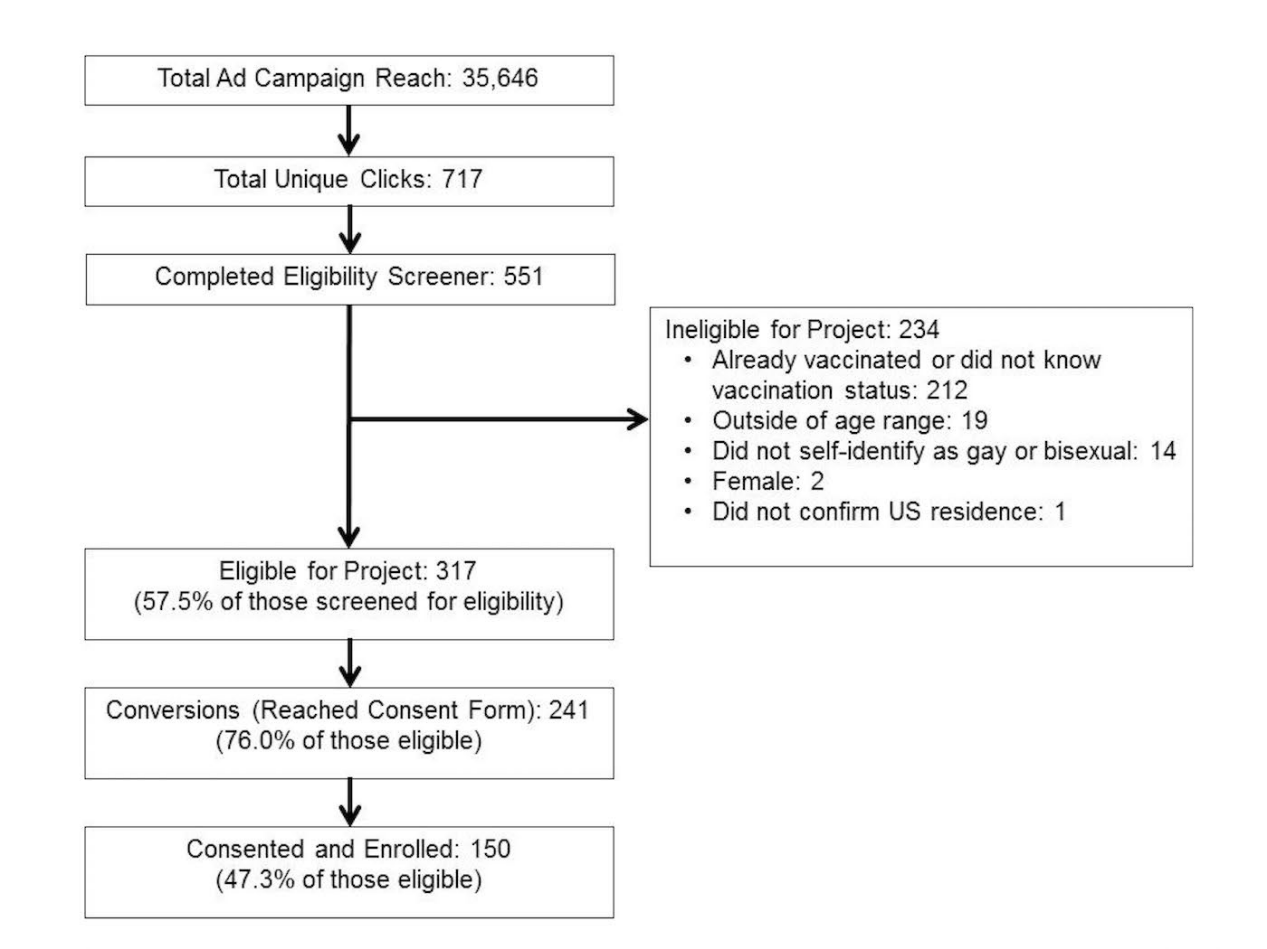
Facebook recruitment campaign results for Outsmart HPV (human papillomavirus).

### Measures

Facebook provided standard data on several metrics related to ad performance. Similar to past studies involving Facebook recruitment campaigns [17,20,25-27], we examined 3 metrics as outcomes: (1) reach (ie, the number of Facebook users an ad was shown to), (2) unique click-through rate (UCTR; number of unique Facebook users who clicked on an ad divided by reach), and (3) conversion rate (number of unique Facebook users who reached the study consent form divided by the number of times an ad was viewed). We express UCTRs and conversion rates as percentages for the remainder of this paper. We also report information on the cost of the recruitment campaign, as well as the demographic and health-related characteristics of enrolled participants (using data from the project eligibility screener and preintervention survey).

### Data Analysis

We calculated descriptive statistics for all Facebook metrics and survey data. We used multivariable Poisson models to determine the effects of our experimental factors (image type, content focus, and disease framing) on ad reach, UCTR, and conversion rate. The multivariable models included all three experimental factors and controlled for recruitment wave. Poisson models for UCTR and conversion rate included an offset or exposure term due to differences in the denominators of these quantities. The analytic dataset used for Poisson analyses included 64 observations (32 created ads that were used in two recruitment waves). We report incidence rate ratios (IRRs) and 95% CI from the multivariable Poisson models. Robust sandwich standard errors were used in calculating the 95% CIs and *P* values. We conducted analyses using Stata version 12.0 (Stata Corp), and all statistical tests were 2-tailed with a critical alpha of .05.

## Results

### Recruitment Campaign Results

Our Facebook recruitment campaign reached a total of 35,646 users who viewed ads for 36,395 times (Figure 2). There were 717 unique clicks on the ads, resulting in an overall UCTR=2.01% (717/35,646). A total of 551 potential participants completed the project eligibility screener, of whom 317 (57.5%) were eligible for participation in the study. Among those found to be ineligible, reasons for ineligibility (more than one reason could apply per person) included: already received HPV vaccine or did not know vaccination status (90.5%, 212/234), outside of the project’s age range (8.1%, 19/234), did not self-identify as gay or bisexual (6.0%, 14/234), female gender (0.9%, 2/234), and did not confirm US residence (0.4%, 1/234).

The recruitment campaign resulted in 241 conversions (76.0% of potential participants found to be eligible [241/317]) and an overall conversion rate of 0.66% (241/36,395). A total of 150 individuals provided consent and were enrolled into the project (47.3% of potential participants found to be eligible [150/317]). It took about 4 days in July 2016 (ie, recruitment wave 1) and about 2 days in September 2016 (ie, recruitment wave 2) to reach this enrollment goal. The total cost of the campaign was US $413.72. This translates into a cost of US $0.58 per unique click on an ad, US $1.72 per conversion, and US $2.76 per enrolled participant.

### Factorial Ad Experiment

The image included in ads affected ad performance. Reach was higher for ads that included an image of a couple (IRR=4.91, 95% CI 2.68-8.97) or a group (IRR=2.65, 95% CI 1.08-6.50) compared with those that included an image of a single person (Table 1). Ads that included an image of a couple also had a higher conversion rate (IRR=2.56, 95% CI 1.13-5.77) and UCTR (OR=1.66, 95% CI 0.99-2.80) than ads that included an image of a single person, though the latter association was of borderline statistical significance in the multivariable model (*P*=.06). For disease framing, ads with main text mentioning an STD had a higher UCTR compared with ads with main text mentioning cancer (IRR=1.34, 95% CI 1.06-1.69). The content focus of ads did not affect ad performance.

**Table 1:** Results of Facebook advertisement factorial experiment. The analytic dataset included 64 observations (32 created ads that were used in two recruitment waves). Multivariable models included all variables in the table and controlled for recruitment wave.

Factor			Reach^a^			UCTR^b,c^			Conversion rate^c,d^		
		n	Mean	IRR^e^ (95% CI)	*P*	Mean	IRR (95% CI)	*P*	Mean	IRR (95% CI)	*P*
**Image type**												
	Single person	16	210.88	ref^f^		1.51	ref		0.35	ref	
	Couple	16	1034.56	4.91 (2.68-8.97)	<.001	2.52	1.66 (0.99-2.80)	.06	0.85	2.56 (1.13-5.77)	.02
	Group	16	557.94	2.65 (1.08-6.50)	.03	1.73	1.03 (0.62-1.71)	.91	0.59	1.76 (0.77-4.03)	.18
	Doctor	16	424.50	2.01 (0.88-4.62)	.10	1.40	0.91 (0.50-1.67)	.77	0.45	1.26 (0.52-3.09)	.61
**Content focus**												
	HPV^g^	32	558.34	ref		2.05	ref		0.62	ref	
	HPV vaccine	32	555.59	1.00 (0.53-1.86)	.99	1.97	0.98 (0.79-1.22)	.86	0.71	1.15 (0.93-1.43)	.19
**Disease framing**													
	Cancer	32	528.38	ref		1.79	ref		0.68	ref	
	STD^h^	32	585.56	1.11 (0.59-2.07)	.75	2.21	1.34 (1.06-1.69)	.01	0.65	0.98 (0.79-1.22)	.85

^a^Reach was defined as number of Facebook users ad was shown to.

^b^Unique click-through rate (UCTR) was defined as the number of unique users who clicked on an ad divided by reach.

^c^Displayed as a percentage.

^d^Conversion rate was defined as the number of unique users who reached the study consent form divided by the number of times an ad was viewed.

^e^IRR: incidence rate ratio.

^f^ref: reference group.

^g^HPV: human papillomavirus.

^h^STD: sexually transmitted disease.

### Participant Characteristics

Enrolled participants were from 31 states and the District of Columbia. Most participants were in the age range of 22-25 years (58.7%, 88/150), non-Hispanic white (56.7%, 85/150), and not married or living with a partner (80.0%, 120/150; Multimedia Appendix 1). About 82.7% (124/150) of participants self-identified as gay. Most participants did not have a college degree (62.7%, 94/150) and reported a household income of less than US $50,000 (76.0%, 114/150). Most participants had some form of health insurance (82.0%, 123/150), though fewer than half reported having a routine medical check-up in the last year (46.7%, 70/150). About half of the participants (51.3%, 77/150) reported they were younger than 18 years of age at sexual debut (first vaginal, anal, or oral intercourse), and most reported having at least six male sexual partners during their lifetime (64.7%, 97/150). Few participants reported being positive for human immunodeficiency virus (HIV; 5.3%, 8/150) or a history of either genital warts (6.0%, 9/150) or another STD (18.0%, 27/150).

## Discussion

### Principal Findings

Reaching and recruiting young gay and bisexual men may be a challenge faced by interventions to increase HPV vaccination. Social media sites represent a potentially effective strategy for overcoming this challenge, yet little is known about the effectiveness of this strategy in recruiting for such interventions. We successfully enrolled 150 eligible young gay and bisexual men via Facebook ads for a pilot test of Outsmart HPV. Recruitment occurred over a short time period and cost less than US $500 total, translating into less than US $3 spent on recruitment per enrolled participant. The recruitment metrics for our study (eg, UCTR and cost per enrolled participant) are similar to those from past studies that recruited via Facebook ads [16,17,25,29]. Our findings not only add to the growing body of literature that shows Facebook ads are a convenient and cost-efficient recruitment strategy for research studies but specifically suggest that they are an effective strategy for recruiting young gay and bisexual men for Web-based HPV vaccination interventions.

Findings from our study also identify potential strategies for optimizing the performance of Facebook ads. Similar to a past study [27], ad images had a meaningful effect on ad performance. Ads that featured an image of a young adult male couple, and to a lesser degree an image of a group of young adult men, performed better than ads that featured a single young adult male. Ads that included an image of a couple may have appealed to the importance of romantic relationships during adolescence and young adulthood [30]. Furthermore, these ads could have prompted potential participants to think about the health of their sexual partners, which may have been motivating since previous research suggests that young sexual minority men view the protection of their partners’ health as an advantage of HPV vaccination [31]. Ads that included an image of a group of young adult men may have affected potential participants’ perceived social norms about the topic and the study. Positive social norms have been previously associated with HPV vaccination behaviors among young gay and bisexual men [7]. Future studies that recruit through Facebook should use ads that feature multiple individuals in the target age range to help improve ad performance and recruitment. Furthermore, we recommend that recruitment ads in future research feature individuals who are racially or ethnically diverse, as we did in this study, so that the ads are relatable and appealing to a wide audience.

The text included in Facebook ads also affected ad performance in our study. Ads with main text mentioning an STD had a higher UCTR compared with ads mentioning cancer. Past research suggests that adolescents and young adults are less future-orientated (eg, tend to focus on potential short-term vs long-term consequences) than older adults [32], and potential participants likely perceived STDs to be a more relevant and immediate health outcome than cancer. Indeed, nearly half of all incident STDs in the United States occur among adolescents and young adults [33], whereas cancer is much less common among young adults than older ages [34]. Our finding is also consistent with past research examining the effects of message framing on HPV vaccine acceptability, as young adults tended to be more receptive to HPV vaccine when it was presented in the context of an STD or genital warts [35,36]. This is in contrast to older adults, who tended to be more receptive to HPV vaccine when it was presented as preventing cancer [37,38]. It is therefore important that future efforts consider framing HPV and HPV vaccine in the context of an STD when targeting materials including study recruitment materials, toward young adults.

The characteristics of participants in our study were comparable with past samples of gay and bisexual men who were recruited through various other strategies. For example, the demographic characteristics of participants in our study (eg, age, race or ethnicity, education level) are highly similar to those of young sexual minority men from the National Survey of Family Growth and the National Longitudinal Study of Adolescent Health [39,40]. Furthermore, about 80% of participants in our study self-identified as gay, which closely resembles other national samples of gay and bisexual men [7,41-43]. Health-related characteristics (eg, lack of health insurance and receipt of routine medical check-up in the last year) were also similar between participants in our study and those from past studies [7,40,44,45]. These similarities should not be altogether surprising given the ubiquitous nature of Facebook use among young adults [46], and they suggest that Facebook ads can recruit diverse samples of young gay and bisexual men that are comparable with what would be obtained through more traditional recruitment methods. Future research should further explore these comparisons, as well as continue to monitor how samples of young gay and bisexual men recruited via Facebook compare with those recruited through other social media sites that may have different audiences (eg, Instagram, Grindr, and MiGente). Data from the American Men’s Internet Survey suggest that characteristics of sexual minority men differ by the social media site used of recruitment [47], and it is important to continue to monitor these potential differences as social media evolve.

### Strengths and Limitations

Study strengths include the use of an experimental design, recruitment of a national sample, and examining several metrics related to ad performance. Our study also has several limitations. Data were not available on the characteristics of Facebook users who were shown an ad but did not click on it. Similarly, we were not able to assess reasons why Facebook users did not click on an ad or clicked on an ad but did not complete the project eligibility screener. We were also not able to link ad performance data with data from study surveys to determine how ad performance may have differed across demographic groups. Future research should identify strategies for linking these data and explore potential differences in ad performance. Fraudulent accounts (eg, multiple accounts) are a growing concern for Web-based research [48], and we used several recommended strategies to reduce this possibility. We used a Completely Automated Public Turing test to tell Computers and Humans Apart (CAPTCHA) during the project account creation process, inspected participants’ account and contact information (eg, email address) for similarities between accounts, and inspected survey data for inconsistent or illogical responses [48]. Our pilot test had a somewhat modest sample size, and future efforts should examine how ad performance and metrics may change over time when recruiting large samples of young gay and bisexual men.

### Conclusions

Facebook ads are a convenient and cost-efficient strategy for reaching and recruiting young gay and bisexual men for a Web-based HPV vaccination intervention. Importantly, the characteristics of young gay and bisexual men enrolled via this recruitment strategy are similar to those enrolled via other recruitment strategies. Future Facebook recruitment efforts for this population should strongly consider the importance of ad content. Specifically, it may be beneficial to include ads that feature a young adult male couple and that frame HPV and HPV vaccine in the context of an STD.

## Appendix

Multimedia Appendix 1 Demographic and health-related characteristics of young gay and bisexual men enrolled in Outsmart HPV (human papillomavirus; n=150).

## Abbreviations

ACIP: Advisory Committee on Immunization Practices
HPV: human papillomavirus
CHCR: Center for Health Communications Research
IRR: incidence rate ratio
LGBT: lesbian, gay, bisexual, and transgender
STD: sexually transmitted disease
UCTR: unique click-through rate
