# Supplementary material for: Recruiting Young Gay and Bisexual Men for a Human Papillomavirus Vaccination Intervention Through Social Media: The Effects of Advertisement Content
Source: JMIR Public Health Surveill. 2017 Jun 2;3(2):e33. doi: 10.2196/publichealth.7545 (PMC5473946; doi:10.2196/publichealth.7545)
Supplement: Multimedia Appendix 1 [file publichealth_v3i2e33_app1.pdf]

| | n (%) |
|---|---|
| **Demographic Characteristics** | |
| Sexual identity | |
| Gay | 124 (82.7) |
| Bisexual | 26 (17.3) |
| Age (years) | |
| 18-21 | 62 (41.3) |
| 22-25 | 88 (58.7) |
| Race / ethnicity | |
| White, non-Hispanic | 85 (56.7) |
| African American, non-Hispanic | 20 (13.3) |
| Other race, non-Hispanic | 10 (6.7) |
| Hispanic | 35 (23.3) |
| Marital status | |
| Other | 120 (80.0) |
| Living with partner or married | 30 (20.0) |
| Education level | |
| Some college or less | 94 (62.7) |
| College degree or more | 56 (37.3) |
| Household income | |
| <$50,000 | 114 (76.0) |
| ≥$50,000 | 36 (24.0) |
| Employment status | |
| Not currently employed | 9 (6.0) |
| Currently employed | 87 (58.0) |
| Student | 54 (36.0) |
| | |
| **Health-Related Characteristics** | |
| Health insurance | |
| None | 27 (18.0) |
| Covered by parents' insurance | 67 (44.7) |
| Covered by other insurance | 56 (37.3) |
| Had a routine medical check-up in the last year | |
| No | 80 (53.3) |
| Yes | 70 (46.7) |
| Age at sexual debut[a] (years) | |
| <18 | 77 (51.3) |
| ≥18 | 73 (48.7) |
| Number of male sexual partners during lifetime | |
| 5 or fewer | 53 (35.3) |

| | |
|---|---|
| 6 or more | 97 (64.7) |
| HIV status | |
| Negative | 142 (94.7) |
| Positive | 8 (5.3) |
| History of genital warts | |
| No | 141 (94.0) |
| Yes | 9 (6.0) |
| History of other STD | |
| No | 123 (82.0) |
| Yes | 27 (18.0) |

*Note*. HIV = human immunodeficiency virus, STD = sexually transmitted disease.

[a]Age at first vaginal, anal, or oral intercourse.
